# Inhibition of Apoptotic Pathways Improves DNA Integrity but Not Developmental Competence of Domestic Cat Immature Vitrified Oocytes

**DOI:** 10.3389/fvets.2020.588334

**Published:** 2020-10-16

**Authors:** Martina Colombo, Jennifer Zahmel, Stefanie Jänsch, Katarina Jewgenow, Gaia Cecilia Luvoni

**Affiliations:** ^1^Dipartimento di Scienze Veterinarie per la Salute, la Produzione Animale e la Sicurezza Alimentare “Carlo Cantoni”, Università degli Studi di Milano, Milan, Italy; ^2^Department of Reproduction Biology, Leibniz-Institute for Zoo and Wildlife Research, Berlin, Germany

**Keywords:** apoptosis, cryopreservation, embryo, feline, gamete, TUNEL

## Abstract

Being a model for endangered wild felids, cryopreservation protocols for domestic cat oocytes are under continuous development. Immature vitrified oocytes (VOs) are a valuable resource for fertility preservation programs, but they often degenerate after warming and their *in vitro* development is poor. Since the exact mechanisms are not clear, this study assessed whether vitrification might trigger two apoptotic markers (DNA fragmentation and caspase activity, Experiment I) and the effects of a chemical inhibitor (i.e., the pan-caspase inhibitor Z-VAD-FMK) on the same markers (Experiment II) and on VOs *in vitro* development (Experiment III). The overarching aim was to check whether apoptosis inhibition might be a strategy to improve cat oocytes cryotolerance. In Experiment I, vitrification induced DNA fragmentation and increased caspase activity in VOs incubated for 24 h after warming (DNA fragmentation: 59.38%; caspase activity: 414.6 ± 326.8) compared to a fresh control (9.68%; 199.6 ± 178.3; *p* = 0.02). In Experiment II, the addition of Z-VAD-FMK to vitrification-warming and incubation media decreased DNA fragmentation and caspase activity (8.82%; 243.7 ± 106.9) compared to control (untreated) VOs (69.44%; 434.5 ± 248.3; *p* < 0.001). In Experiment III, Z-VAD-FMK brought maturation rates of treated VOs close to those of fresh oocytes (53.13 and 65.38%, respectively, *p* = 0.057), but there were no differences in VOs embryo development (cleavage rates; Z-VAD-FMK-treated VOs: 34.38%; control VOs: 31.78%; *p* = 0.69). In summary, vitrification increased apoptotic markers in cat VOs, and while Z-VAD-FMK was able to hinder DNA damage and caspase activity, its addition was not determinant for embryo development. To make the best use of VOs, other oocyte *in vitro* maturation and embryo culture strategies, such as the addition of other inhibitors or their prolonged use, should be investigated.

## Introduction

Cryotolerance of cat oocytes is suboptimal. Since the domestic cat is used as a model for the development of cryopreservation strategies transferable to its wild relatives (1), survival and development of feline oocytes after vitrification and warming are critical steps to make gamete biobanks worth the effort. Immature vitrified oocytes (VOs) experience several cryoinjuries, which affect their ability to resume meiosis and develop properly into embryos after warming. Both temperature decrease and high concentrations of cryoprotectants have a role in inducing damages. The most commonly reported are oxidative stress, cumulus cells loss, zona pellucida hardening, and cytoskeleton damages, which lead to disassembly of the meiotic spindle and DNA alterations (2, 3), but the exact mechanisms for their poor *in vitro* survival and development are still unknown (4).

In several species and cell types, including gametes, cryopreservation-induced apoptosis has been reported (5–14). In domestic cat immature oocytes, vitrification affects mitochondria aggregation (15), and probably also the permeability of gap junctions hemichannels, causing the loss of small essential metabolites, ionic imbalance, and the penetration of small, potentially toxic, molecules (16). However, information is still limited, and only one study tried to target the apoptosis pathways through an inhibitor of a signaling molecule known as ROCK (Rho-associated coiled-coil containing protein kinase), obtaining an improvement in the developmental competence of VOs in terms of normal fertilization and cleavage rates (4).

In other species, several strategies have been experimented on VOs to improve their outcomes, with encouraging results. Besides the addition of antifreeze proteins (17, 18) or the inhibition of factors regulating signal transduction (19), the use of apoptosis inhibitory molecules was demonstrated to be applicable and effective in pig vitrified oocytes (14). These molecules (e.g., Z-IETD-FMK, Z-LEHD-FMK, Z-VAD-FMK) block caspases, which are the effector enzymes involved in proteolysis and DNA fragmentation during apoptosis, and thus they hamper cell death. One of these (i.e., the tripeptide Z-VAD-FMK, Benzyloxycarbonyl-Val-Ala-Asp Fluoromethyl ketone) is a broad-spectrum, cell-permeable caspase inhibitor, which was used successfully in reversing cryopreservation-induced apoptotic degeneration of porcine (20) and bovine embryos (21) and of different somatic cells (22, 23).

This study aimed to investigate whether cat VOs cryotolerance can be improved by the inhibition of apoptotic pathways. For this purpose, we assessed whether vitrification might trigger two apoptotic markers (DNA fragmentation and caspase activity, Experiment I) and the effects of a chemical inhibitor (i.e., the pan-caspase inhibitor Z-VAD-FMK) on the same markers (Experiment II) and on VOs developmental competence (Experiment III).

## Materials and Methods

### Chemicals and Reagents

All chemicals and reagents were purchased from Sigma-Aldrich (St. Louis, MO, USA), unless otherwise stated.

### Experimental Design

Three experiments were performed to investigate the effect of vitrification of immature domestic cat oocytes on some apoptosis markers and whether cat VOs cryotolerance and *in vitro* development can be improved with the use of a pan-caspase inhibitor. Its influence on the same apoptosis markers and on the *in vitro* development of cat VOs was assessed.

In Experiment I, to check whether Cryotop vitrification induces the activation of apoptotic pathways in cat VOs, fresh (FOs, negative control), hydrogen peroxide-exposed (HPOs, positive control), and vitrified-warmed oocytes were analyzed by TUNEL assay (to detect DNA fragmentation) and by a caspase activity assay. Since degeneration might start slowly after warming, VOs were analyzed after 2 [2hVOs, based on (24)] or 24 h (24hVOs, standard length of cat oocytes *in vitro* maturation—IVM) incubation.

Experiment II was aimed to investigate the influence of a pan-caspase inhibitor on the same apoptosis markers (i.e., DNA fragmentation, caspase activity) analyzed in Experiment I; Z-VAD-FMK was added to vitrification-warming media only [VOs(+/−)] or to both vitrification-warming media and post-warming incubation medium [VOs(+/+)]. The length of the post-warming incubation (24 h) was chosen based on the results of Experiment I. Untreated VOs [VOs(−/−)], which were vitrified/warmed and then incubated without Z-VAD-FMK, were used as control.

In Experiment III, the aim was to check the influence of the pan-caspase inhibitor Z-VAD-FMK on the *in vitro* development of cat VOs; the treatment that gave the best results in Experiment II [VOs(+/+)] was used for *in vitro* embryo production to assess maturation and embryo development compared to untreated VOs [VOs(−/−)] and fresh oocytes (FOs). In this Experiment, post-warming incubation corresponded to IVM.

Groups and treatments are described in detail in sections “Staining for apoptotic signal detection (Experiments I and II)” and “*In vitro* maturation”.

### Ovaries and Oocyte Retrieval

Ovaries (*n* = 244) from healthy queens (*Felis catus*) were harvested at random stages of the estrous cycle during routine ovariectomies or ovariohysterectomies. The study did not require an ethical approval because cat ovaries were collected as byproducts from routine surgeries.

After surgery, ovaries were immediately placed in Minimum Essential Medium Eagle HEPES Modification (HEPES-MEM) supplemented with 3 mg/mL bovine serum albumin (BSA) and a mixture of antibiotics and antimycotics (100 IU/mL of penicillin G sodium, 0.1 mg/mL of streptomycin sulfate, 0.25 μg/mL of amphotericin B), and transported to the laboratory at room temperature within 4 h.

Ovaries were minced in PBS with 0.1% (w/v) polyvinyl alcohol to release the oocytes. A surgical scalpel (size 10) was used to cut the ovarian cortex. Only immature COCs with darkly pigmented ooplasm completely surrounded by 3–4 layers of cumulus cells [Grade I (25)] were selected for the experiments.

### Vitrification and Warming of Immature Cumulus-Oocyte Complexes (COCs)

Oocytes were vitrified by the Cryotop method (26, 27), as previously described for cat oocytes (28). Briefly, groups of 4–8 oocytes were equilibrated at room temperature in an equilibration solution containing 7.5% (v/v) ethylene glycol (EG) and 7.5% dimethylsulfoxide (DMSO) in Medium 199, with 20% fetal bovine serum (FBS) for 15 min. Then, they were transferred into a vitrification solution (15% [v/v] EG, 15% DMSO and 0.5 M sucrose in Medium 199 with 20% FBS), placed on Cryotop polypropylene strip, removing excess liquid to reduce the volume as much as possible, and directly immersed into liquid nitrogen in <90 s. Leaving the Cryotop completely immersed in liquid nitrogen, it was capped with the help of some clamps. When vitrification of all the oocytes was completed, Cryotops were transferred in a goblet and kept in a storage tank until warming.

At warming, the Cryotop strip was immersed for 1 min in a thawing solution at 37°C containing 1 M sucrose in Medium 199, with 20% FBS. Vitrified oocytes were retrieved and transferred for 3 min in a solution containing 0.5 M sucrose in Medium 199, with 20% FBS and then for 5 min in a solution without sucrose. Finally, they were washed again in the same solution (Medium 199 with 20% FBS) and then moved to the appropriate medium (Medium 199 for incubation, Experiments I and II; IVM medium, Experiment III).

In Experiments II and III, for Z-VAD-FMK-treated oocytes, the final concentration of Z-VAD-FMK (Z-VAD[OMe]-FMK, MedChemExpress, Monmouth Junction, NJ, USA) in all vitrification-warming solutions and IVM media was 20 μM (14, 20, 21, 29). The stock solution (20 mM) was prepared in DMSO. This concentration was chosen so that DMSO would have been diluted 1:1,000 in the media, with a concentration (0.1% v/v) that was advised by the manufacturer to avoid cell toxicity.

Since the addition of Z-VAD-FMK meant an addition of DMSO, the volume of inhibitor was subtracted from the total amount of DMSO for the preparation of equilibration and vitrification solutions for Z-VAD-FMK-treated VOs. Likewise, an amount of DMSO corresponding to the amount of inhibitor (1:1,000) was added to the solutions for stepwise warming of control VOs. For the same reason, in incubation and IVM media of control VOs (i.e., without Z-VAD-FMK), a corresponding volume of DMSO (1:1,000) was added.

### Staining for Apoptotic Signal Detection (Experiments I and II)

Cell Meter™ TUNEL Apoptosis Assay Kit ^*^Red Fluorescence^*^ (AAT Bioquest, Sunnyvale, CA, USA) and CellEvent™ Caspase-3/7 Green Detection Reagent (Thermo Fisher Scientific—Invitrogen, Monza, Italy) were used to stain the oocytes for DNA fragmentation and caspase activation, respectively, following the manufacturers' instructions. Briefly, oocytes were incubated for 1 h at 38.5°C in TUNEL and caspase dyes. Then, they were denuded and counterstained with Hoechst 33342 (0.01 mg/mL) to identify the nucleus. Decumulation was performed after incubation in the dyes to avoid a possible influence of pipetting-derived shear stress on cell apoptosis (30). Finally, the slides were covered with an antifade reagent (Fluoromount™ Aqueous Mounting Medium). Dyes maximum excitation/emission wavelengths were as follows:

– TUNEL Assay: 556/579 nm;
– Caspase Detection Reagent: 502/530 nm;
– Hoechst: 352/461 nm.

**Figure 1:**
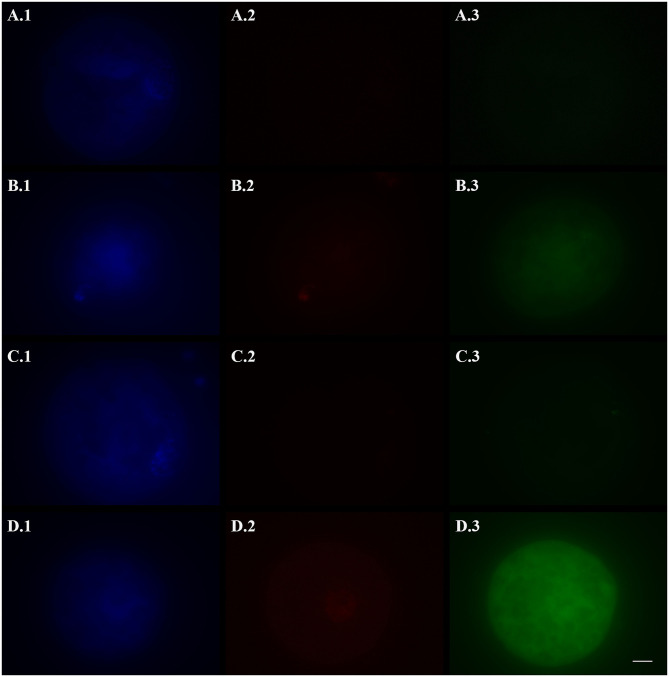
Representative fluorescence micrographs of vitrified **(A,B)**, fresh (**C**, negative control), and hydrogen peroxide-treated (**D**, positive control) domestic cat oocytes stained with Hoechst 33342 **(1)**, TUNEL assay **(2)**, and a caspase activity assay **(3)** to assess the activation of apoptotic pathways. Vitrified oocytes were stained 2 (A) or 24 (B) h after warming. Bright blue fluorescence **(A.1,B.1,C.1,D.1)** indicates the nuclear material. Bright red fluorescence **(B.2,D.2)** in the nuclear area indicates DNA fragmentation by TUNEL assay. Green fluorescence in the ooplasm **(A.3,B.3,C.3,D.3)** indicates, according to its intensity, the extent of caspase activity. Images were captured in black and white and pseudo-colored after acquisition with the Imaging Software ZEN 2.5 blue edition. Black and white balance of Hoechst (1) and TUNEL (2) images was adjusted after coloring to make nuclear stainings more visible in print. Caspase images, which were used for quantification, were not modified. Scale bar: 20 μm.

**Figure 2:**
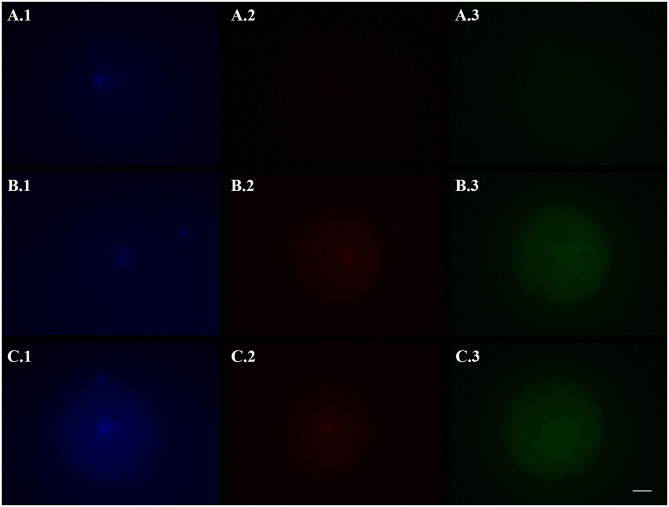
Representative fluorescence micrographs of domestic cat vitrified oocytes stained 24 h after warming with Hoechst 33342 **(1)**, TUNEL assay **(2)**, and a caspase activity assay **(3)** to assess the activation of apoptotic pathways. **(A)** Oocytes vitrified-warmed and incubated for 24 h in the presence of the pan-caspase inhibitor Z-VAD-FMK; **(B)** Oocytes vitrified-warmed in the presence of Z-VAD-FMK and incubated for 24 h without it; **(C)** Oocytes vitrified-warmed and incubated for 24 h without Z-VAD-FMK (control). Bright blue fluorescence **(A.1,B.1,C.1)** indicates the nuclear material. Bright red fluorescence **(B.2,C.2)** in the nuclear area indicates DNA fragmentation by TUNEL assay. Green fluorescence in the ooplasm **(A.3,B.3,C.3)** indicates, according to its intensity, the extent of caspase activity. Images were captured in black and white and pseudo-colored after acquisition with the Imaging Software ZEN 2.5 blue edition. Black and white balance of Hoechst (1) and TUNEL (2) images was adjusted after coloring to make nuclear stainings more visible in print. Caspase images, which were used for quantification, were not modified. Scale bar: 20 μm.

Slides were evaluated under a fluorescence microscope (Axiovert 100, Zeiss) equipped with a digital camera (AxioCam MRc5, Zeiss) at 400 × magnification to count the number of TUNEL-positive oocytes (showing a bright red fluorescence in nucleus area) and to esteem caspase activity (i.e., green fluorescence) with the fluorescence intensity mean value of ooplasm, measured with the Imaging Software ZEN 2.5 blue edition (Carl Zeiss Microscopy). Fluorescence values were corrected for background fluorescence. Oocytes whose nucleus could not be identified by Hoechst staining were excluded by the analysis (for Experiment I: one oocyte in 24hVOs and one in FOs were discarded; for Experiment II: three oocytes in VOs[+/+], one in VOs[+/−], and one in VOs[−/−] were discarded; no significant differences among groups in the number of nucleus-missing oocytes; Fisher's *p* = 0.358). Images were captured in black and white and under the same settings. Figures 1, 2 shown in the present paper were pseudo-colored after image acquisition.

In Experiment I, four experimental groups were compared. Fresh oocytes (FOs, negative control) were stained right after collection. In positive control oocytes (HPOs), apoptosis was induced with a 3 h incubation in 100 μM H_2_O_2_ in Medium 199 (31, 32) before staining. Vitrified oocytes were incubated after warming for 2 h (2hVOs) (24) or 24 h (24hVOs) in Medium 199 to assess whether the activation of apoptotic pathways might occur soon after warming or slowly during the following incubation time.

In Experiment II, three experimental groups of VOs were compared. Based on the results of Experiment I, warmed oocytes were incubated for 24 h before staining. Control VOs [VOs(−/−)], which were vitrified-warmed without Z-VAD-FMK, were incubated in plain Medium 199. Oocytes vitrified-warmed with the addition of Z-VAD-FMK, instead, were either incubated in plain Medium 199 [VOs(+/−)] or in Medium 199 with the addition of 20 μM Z-VAD-FMK [VOs(+/+)].

### *In vitro* Embryo Production (Experiment III)

#### *In vitro* Maturation

Based on the results of Experiment II, three experimental groups underwent IVM to assess the developmental competence of VOs treated with a pan-caspase inhibitor:

– Treated VOs [VOs(+/+)], i.e., oocytes vitrified-warmed and *in vitro* matured with the addition of Z-VAD-FMK.
– Control VOs [VOs(−/−)], i.e., oocytes vitrified-warmed and matured in standard conditions.
– Fresh oocytes (FOs), as system control.

Oocytes were singly *in vitro* matured for 24 h in a controlled atmosphere (39°C and 5% CO_2_ in air) in 20 μL microdrops of IVM medium (33, 34) [Medium 199 supplemented with 3 mg/mL BSA, 0.1 mg/mL cysteine, 1.4 mg/mL HEPES, 0.25 mg/mL sodium pyruvate, 0.6 mg/mL sodium lactate, 0.15 mg/mL L-glutamine, 0.055 mg/mL gentamicin, 0.2 IU/mL human luteinizing hormone (LH), and 0.5 IU/mL human pituitary follicle-stimulating hormone (hFSH)] covered by mineral oil in Petri dishes.

#### Epididymal Sperm Recovery and *in vitro* Fertilization (IVF)

*In vitro* fertilization was performed with fresh feline epididymal spermatozoa obtained after routine orchiectomy of adult tomcats (*n* = 10). Testes were maintained at 4°C without any medium for up to 24 h before processing. The epididymides were dissected from isolated testicles and placed in a Petri dish in HEPES-buffered Medium 199. Spermatozoa were obtained from *vas deferens* and *cauda epididymis* by slicing with scissors at room temperature, and flushing sperm suspension through a 30 μm filter (Sysmex Partec GmbH, Görlitz, Germany). Subjective motility and concentration by Bürker chamber were determined.

After 24 h of IVM, oocytes were removed from the IVM dishes, washed twice, and transferred into 50 μL microdrops of fresh IVF medium (34) (Medium 199 supplemented with 3 mg/mL BSA, 0.1 mg/mL cysteine, 1.4 mg/mL HEPES, 0.25 mg/mL sodium pyruvate, 0.6 mg/mL sodium lactate, 0.15 mg/mL L-glutamine, 0.055 mg/mL gentamicin, and 2.2 IU/mL heparin) covered by mineral oil in Petri dishes. Diluted spermatozoa, previously centrifuged (500 g, 5 min) and resuspended in IVF medium, were added to the microdrops containing the oocytes to reach 100 μL volume with a final concentration of 1 × 10^6^ motile spermatozoa/mL. Each IVF drop contained 7–11 oocytes from the same experimental group. Oocytes and spermatozoa were co-incubated in a controlled atmosphere (39°C and 5% CO_2_ in air) for 18 h.

#### *In vitro* Embryo Culture

After IVF, all oocytes were gently washed in *in vitro* culture (IVC) medium (Ham's F-10 supplemented with 5% FBS, 0.11 mg/mL sodium pyruvate, 0.075 mg/mL L-glutamine, 0.6 mg/mL gentamicin) to remove spermatozoa and residual cumulus cells with the help of a stripper micropipette (The Stripper, BioTipp, Waterford, Ireland) equipped with 155 and 125 μm tips (RI-Tip, Gynemed, Lensahn, Germany). Presumptive embryos were moved to 20 μL microdrops of IVC medium covered by mineral oil in Petri dishes, where they were cultured singly for up to 8 days in a controlled atmosphere (39°C, 5% CO_2_, and 5% O_2_). The medium was not changed during embryo culture. During IVC, assessment of embryo development was performed every 24 h under an inverted microscope at 200 × magnification (Axiovert 100, Zeiss).

#### Assessment of Maturation, Fertilization, and Embryonic Developmental Rates

Two days after IVF, uncleaved oocytes were deprived of remaining cumulus cells and bound spermatozoa by mechanical displacement with a stripper micropipette equipped with a 125 μm tip, placed on a slide, air-dried, and fixed in 80% ethanol overnight at 4°C. Chromatin configuration was then evaluated by propidium iodide (PI; Thermo Fisher Scientific) staining (1 mg/mL, 1:100 in PBS). Growing embryos were cultured until day 8 or until they showed signs of degeneration and then fixed and stained with PI to ascertain their developmental stage based on the number of blastomere nuclei. Maturation stage (metaphase II, MII) in unfertilized oocytes was identified by the presence of a tightly packed group of chromosomes in the form of the first polar body, and another group that were well spread, allowing for the identification of individual chromosomes (35).

Nuclear evaluation was performed under a fluorescence microscope (Axiovert 200M, Zeiss) at 200 × magnification, considering that the PI maximum excitation and emission wavelengths are 535 and 617 nm, respectively.

The total number of *in vitro* matured oocytes was calculated as the sum of unfertilized MII oocytes, fertilized oocytes (i.e., uncleaved but showing pronuclei) and cleaved embryos; accordingly, the number of fertilized oocytes was calculated as the sum of fertilized oocytes and cleaved embryos. For the assessment of embryonic development, cleaved embryos (2–4 cells), 5–8 cells, 9–16 cells, morulae, and blastocysts stages were recorded.

### Statistical Analysis

In Experiments I and II, TUNEL data were analyzed by Fisher's exact test, whereas caspases activation (i.e., fluorescence intensity) was analyzed by Kruskal-Wallis non-parametric one-way ANOVA followed by Dwass-Steel-Critchlow-Fligner pairwise comparisons (data were not normally distributed by Shapiro-Wilk test; *p* < 0.001 for Experiment I, *p* = 0.005 for Experiment II). In Experiment III, maturation, fertilization, and embryo development rates were analyzed by Fisher's exact test. Analysis were performed with the software *jamovi* (Version 1.0.7.0). The level of significance was set at *p* < 0.05.

## Results

### Experiment I

The activation of apoptotic pathways in fresh and vitrified oocytes is reported in Table 1, and representative pictures are shown in Figure 1. DNA fragmentation (TUNEL-positive oocytes) was the lowest in FOs (negative control) and 2hVOs (*p* = 0.106). The number of oocytes with fragmented DNA increased in 24hVOs compared to 2hVOs (*p* = 0.023), even if 24hVOs still had lower DNA fragmentation than HPOs (positive control, *p* = 0.004). Caspase activity was higher in VOs and HPOs than in FOs (*p* = 0.001), and there were no differences between VOs incubated for different time after warming (2hVOs vs. 24hVOs, *p* = 0.989) or between VOs and HPOs (*p* = 0.766).

**Table 1:** Activation of apoptotic pathways in vitrified and fresh domestic cat oocytes, assessed as DNA fragmentation (TUNEL-positive oocytes) and caspase activity (3 replicates).

**Oocytes**	**n**.	**DNA fragmentation** **% (n.)**	**Caspase activity** **Fluorescence intensity mean value ± SD**
2hVOs	31	29 (9)^a^	321.7 ± 212.3^a^
24hVOs	32	59.4 (19)^b^	414.6 ± 326.8^a^
FOs	31	9.7 (3)^a^	199.6 ± 178.3^b^
HPOs	33	90.9 (30)^c^	420.1 ± 346.1^a^

Different superscripts (a, b, c) within the same column indicate significant differences among groups (p < 0.05).

*2hVOs, vitrified oocytes incubated in Medium 199 for 2 h after warming; 24hVOs, vitrified oocytes incubated in Medium 199 for 24 h after warming; FOs, fresh oocytes (negative control); HPOs, oocytes treated with hydrogen peroxide to induce apoptosis (positive control)*.

### Experiment II

Based on the results of Experiment I, in which 24hVOs had a higher DNA damage, and since the standard length of post-warming incubation in VOs is 24 h (i.e., IVM length for cat oocytes), this time was chosen for incubation to assess the effect of an apoptosis inhibitor. Results of the use of Z-VAD-FMK during vitrification-warming or during vitrification-warming and incubation on DNA fragmentation and caspase activity are reported in Table 2. Representative pictures are shown in Figure 2. Compared to the control [VOs(−/−)], Z-VAD-FMK-treated VOs had lower percentages of TUNEL-positive oocytes (*p* = 0.010), especially those both vitrified-warmed and incubated in the presence of the inhibitor [VOs(+/+)], in which more oocytes had intact DNA than in the other groups (*p* = 0.005). Caspase activity was the lowest in VOs(+/+) (*p* < 0.001), while it did not differ between VOs(+/−) and VOs(−/−) (*p* = 0.588).

**Table 2:** Activation of apoptotic pathways in domestic cat vitrified oocytes (VOs), vitrified-warmed and/or incubated after warming with the addition of the pan-caspase inhibitor Z-VAD-FMK, assessed as DNA fragmentation (TUNEL-positive oocytes) and caspase activity (3 replicates).

**Oocytes**	**n**.	**DNA fragmentation** **% (n.)**	**Caspase activity** **Fluorescence intensity mean value ± SD**
VOs(+/+)	34	8.8 (3)^a^	243.7 ± 106.9^a^
VOs(+/−)	37	37.8 (14)^b^	653.9 ± 591.6^b^
VOs(−/−)	36	69.4 (25)^c^	434.5 ± 248.3^b^

Different superscripts (a, b, c) within the same column indicate significant differences among groups (p < 0.05).

*VOs(+/+), oocytes vitrified-warmed and incubated for 24 h with the addition of Z-VAD-FMK; VOs(+/−), oocytes vitrified-warmed with the addition of Z-VAD-FMK and incubated for 24 h in plain Medium 199; VOs(−/−), oocytes vitrified-warmed and incubated for 24 h in Medium 199-based media without the addition of Z-VAD-FMK (control)*.

### Experiment III

Based on the results of Experiment II, VOs vitrified-warmed and incubated (i.e., *in vitro* matured) for 24 h with Z-VAD-FMK [VOs(+/+)] were chosen for the *in vitro* embryo production to assess their developmental ability compared to control VOs(−/−) and FOs. Maturation, fertilization, and embryo development rates are summarized in Tables 3, 4. As expected, maturation rates of control VOs(−/−) were lower than those of FOs (*p* = 0.004), while VOs(+/+) had similar rates to both VOs(−/−) (*p* = 0.384) and FOs (*p* = 0.057). In addition, fertilization did not differ among the three groups (*p* = 0.082), as well as cleavage/MII ratios (*p* = 0.294). At later stages, embryo development rates did not differ between VOs(−/−) and VOs(+/+) (*p* = 0.226 for 5–8 cells stage), but they were always higher in FOs (*p* = 0.001). This was especially true for late embryonic developmental stages, which are really challenging to reach for VOs, as confirmed by the inability of both VOs(−/−) and VOs(+/+) to form blastocysts.

**Table 3:** Maturation, fertilization, and degeneration rates of vitrified and fresh domestic cat oocytes following *in vitro* embryo production (6 replicates).

**Oocytes**	**n**.	**Maturation^**†**^** **% (n.)**	**Fertilization^**‡**^** **% (n.)**	**Deg/N.A.** **% (n.)**
VOs(+/+)	128	53.1 (68)^a, b^	44.5 (57)^a^	35.2 (45)^a^
VOs(−/−)	129	47.3 (61)^a^	41.1 (53)^a^	38.8 (50)^a^
FOs	130	65.4 (85)^b^	52.3 (68)^a^	13.9 (18)^b^

Different superscripts (a, b) within the same column indicate significant differences among groups (p < 0.05). Percentages calculated on the total number of oocytes in each group.

VOs(+/+), oocytes vitrified-warmed and in vitro matured with the addition of the pan-caspase inhibitor Z-VAD-FMK; VOs(−/−), oocytes vitrified-warmed and matured without the inhibitor Z-VAD-FMK (control); FOs, fresh control oocytes; Deg, degenerated; N.A., not assessable.

† Sum of cleaved embryos, uncleaved fertilized oocytes (i.e., with pronuclei) and unfertilized metaphase II oocytes.

‡ *Sum of cleaved embryos and uncleaved fertilized oocytes*.

**Table 4:** Embryonic developmental rates of vitrified and fresh domestic cat oocytes following *in vitro* embryo production (6 replicates).

**Oocytes**	**Matured n**.	**2–4 cells** **% (n.)**	**5–8 cells % (n.)**	**9–16 cells** **% (n.)**	**Morulae % (n.)**	**Blastocysts** **% (n.)**
VOs(+/+)	68	64.7 (44)^a^	29.4 (20)^a^	8.8 (6)^a^	1.5 (1)^a^	0 (0)^a^
VOs(−/−)	61	67.2 (41)^a^	19.7 (12)^a^	6.6 (4)^a^	1.6 (1)^a^	0 (0)^a^
FOs	85	72.9 (62)^a^	62.4 (53)^b^	49.4 (42)^b^	35.3 (30)^b^	15.3 (13)^b^

Different superscripts (a, b) within the same column indicate significant differences among groups (p < 0.05). Percentages calculated on the total number of matured oocytes in each group.

*VOs(+/+), oocytes vitrified-warmed and in vitro matured with the addition of the pan-caspase inhibitor Z-VAD-FMK; VOs(−/−), oocytes vitrified-warmed and matured without the inhibitor Z-VAD-FMK (control); FOs, fresh control oocytes*.

## Discussion

To overcome the poor *in vitro* maturation and embryonic developmental rates of cat VOs, several studies focused on the modification of vitrification procedures (36, 37), most recently with the addition of follicular fluid extracellular vesicles to vitrification-warming media (38), or on the modification of post-warming conditions, such as *in vitro* culture environments (39, 40). In addition, targeting apoptosis pathways seemed to be beneficial for cat VOs developmental competence (4), and the use of apoptosis inhibitors was successful for vitrified embryos (20, 21) and oocytes (14) in other species. In the present study, we assessed DNA fragmentation and caspase activity with the aim of understanding whether vitrification might activate apoptotic pathways in cat immature oocytes, and we applied the apoptosis inhibitor Z-VAD-FMK to evaluate its effect on these apoptotic markers and on the outcomes of *in vitro* embryo production, with the overarching aim of improving VOs cryotolerance.

Overall, results showed that vitrification of immature cat oocytes induced DNA damage and caspase activation. The addition of the pan-caspase inhibitor Z-VAD-FMK lowered these markers and brought VOs maturation rates close to those of fresh oocytes, even though it did not have any influence on embryo development.

During oocyte apoptosis, some of the main players are caspases. Caspases are proteases, thus enzymes that can cleave other polypeptides, which usually need to be cleaved by other enzymes in order to be activated, often by other caspases in a cascade (41). Caspase activity leads to the wreckage of several cellular components, resulting in some well-defined damages that are typical of apoptosis and not of other types of cell death (e.g., phosphatidylserine exposure, nuclear condensation, and genomic DNA fragmentation) (42, 43). According to their position in the cascade and their function, caspases can be classified as initiators (e.g., caspases 8 and 9) or effectors (e.g., caspases 3, 6, and 7) (41). Activated caspases 3 and 7, which were the ones identified by fluorescent staining in the present study, can cleave several substrates, also resulting in DNA fragmentation (42), which was hereby assessed by TUNEL assay. In Experiment I, caspase activity was higher in VOs than in FOs, regardless of the length of post-warming incubation. After 24 h, also DNA fragmentation was significantly higher in VOs than in FOs, while it was similar between 2hVOs and FOs. Therefore, caspases activity had already started soon after warming, but more time might be necessary for some enzymes to reach the nucleus and start DNA fragmentation to the point it is detectable by TUNEL assay. This might also be related to our previous observations of VOs post-warming degeneration. Survival rates after warming of oocytes vitrified with this protocol are generally higher than 90%, but their viability decreases during the following incubation (40), as also reported in pig Cryotop-vitrified oocytes (24). In other species, several studies witnessed the involvement of apoptotic cell death in VOs post-warming degeneration (10, 13, 14). In the domestic cat, one study focused on immature vitrified oocytes revealed that the expression of apoptosis and DNA repair proteins increased after IVM (44). This agrees with the findings of Experiment I, in which caspase activity and DNA damage were detected at the end of 24 h of incubation.

Therefore, we hypothesized that the partial inhibition of apoptosis, induced by a molecule acting only on some components of the apoptotic cascade, might be beneficial for VOs survival and development. In the present study, Z-VAD-FMK was used during vitrification-warming or during vitrification-warming and the following day of culture. This inhibitor acts by binding to the catalytic sites of caspases and hampering their activity. Results of Experiment II showed that the addition of Z-VAD-FMK only during the vitrification-warming procedure was not enough to avoid DNA damage, while the further addition of Z-VAD-FMK during the following 24 h of incubation could significantly reduce caspase activity and DNA damage, achieving results similar to those of FOs in Experiment I. Similarly, the exposure to specific caspase inhibitors during incubation was beneficial for vitrified porcine oocytes in terms of reduced caspase activity and improved developmental capabilities (14). The difference between VOs(+/+) and VOs(+/−), instead, might be due to the short time of exposure when the inhibitor was added only during the vitrification-warming procedure, which lasted in total slightly more than 25 min, considering that the molecular interactions during the storage in liquid nitrogen are negligible. Therefore, a longer culture with the addition of Z-VAD-FMK was beneficial in hindering the activation of apoptotic pathways, and this treatment was chosen for *in vitro* embryo production in Experiment III.

As previously mentioned, due to several cryoinjuries, cat VOs developmental competence might be impaired, with cleavage rates around 15–30% and poor development after the 8–16 cells stage (45). Cleavage rates of VOs obtained in this study (>30% calculated on the total number of oocytes in each group) are encouraging and suggest that the Cryotop vitrification for immature oocytes, together with the IVM-IVF-IVC protocol used in our laboratory, might be a good combination for the *in vitro* embryo production from cryopreserved feline gametes. However, no difference between VOs(+/+) and VOs(−/−) (control) was found in embryo development, and the progression to later stages of development was poor, especially for morula (about 1.5% in both groups) and blastocyst (0%) stages. On the other hand, Z-VAD-FMK benefitted IVM outcomes, whose results were similar to those of FOs. The reason might be that the diminished apoptotic activity observed during IVM, linked to the presence of the inhibitor, did not prevent later degeneration (i.e., after IVF). In other species, the use of different apoptosis inhibitors in vitrification-warming and/or incubation media was effective to increase the cryotolerance of cryopreserved oocytes (14) and embryos (20, 21), leading to higher survival and embryonic developmental rates. In the cat, a molecule that targets other components of the apoptotic pathways was successful in improving embryo development (4), but in this study the use of Z-VAD-FMK did not give the same results. Indeed, ROCK, i.e., a kinase, activated by caspase 3 during apoptosis (46), which is also involved in cytoskeleton organization and therefore in oocyte viability and competence (47), was blocked by its inhibitor ROCKi in (4). In this study, caspases, which are mostly involved in cell death and inflammation, were broadly inhibited by Z-VAD-FMK. This difference might explain the divergent results of ROCKi and Z-VAD-FMK on cat VOs. Other strategies should be experimented in order to improve VOs developmental competence. Chemical inhibitors against different targets could be tested to assess whether their addition during IVM might exert beneficial and long-lasting effects. Another possibility, because of the small improvement Z-VAD-FMK gave during IVM, could be the use of apoptosis inhibitors for the whole *in vitro* embryo production, so also during IVC. During culture, embryos that are not going to develop further tend to arrest their cellular divisions and start degenerating. Thus, the presence of apoptotic inhibitors might hinder this process, and there is evidence that one improved embryo production when added to culture medium (29). However, other strategies should also be planned. Apoptosis is a natural process occurring during development, also in growing embryos, to support the selection of healthy blastomeres during embryo development (48), and we do not know what kind of alterations may arise following apoptosis inhibition, or if it may harm the embryo somehow. In addition, there is still no knowledge about the survival and development of gametes and embryos treated with apoptosis inhibitors once they are transferred to a recipient animal to, hopefully, generate offspring.

Besides the effects of the inhibitor, in the present study we observed that fertilization rates, as well as cleavage/MII ratios, did not differ among VOs and FOs, confirming that cat VOs do not usually undergo zona pellucida hardening [a well-known cryodamage in cryopreserved oocytes (3)], and IVF, rather than intracytoplasmic sperm injection (ICSI), is a suitable technique for their fertilization. In addition, the ability to be fertilized and cleave belongs to all matured oocytes in the same way, whether they were previously vitrified or not.

It was also interesting to notice that a decrease in apoptotic markers did not mean an increase in developmental competence. Since we do not have a threshold to discriminate healthy and dying cells and neither the use of fluorescent staining nor other methods (e.g., immunohistochemistry, RT-PCR) supply an absolute quantification of caspase activity, the evaluation of fragmented DNA might be a better esteem of the number of oocytes, which will be able to be fertilized and undergo cellular divisions. Even if VOs(+/+) and VOs(−/−) showed DNA fragmentation and caspase activity at different extents (Experiment II), embryo development (Experiment III) was equally poor. Much more than DNA integrity is needed to accomplish embryo formation, and probably here lies the reason for impaired VOs development. Another possibility might be that VOs undergo necroptosis [i.e., another type of programmed cell death, independent from caspases and also known as inflammatory cell death (49)] due to the exposure to Z-VAD-FMK and its inhibitory effect on caspase 8 (49). If this was the case, the absence of an increase in apoptotic markers would not be indicative of oocyte health, and the poor embryo development might be due to a non-apoptotic degeneration.

In the field of oocyte cryopreservation, research concerning the improvement of the efficiency of *in vitro* embryo production should give more attention to the progression of embryo development after oocyte fertilization and the first cellular divisions. In the growing embryo, cellular divisions are complex and well-orchestrated processes (50). Among the event allowing each division, DNA duplication and cytoskeleton dynamic are fundamental, as well as maternal mRNAs, but unfortunately all of these might be damaged in cryopreserved cells (3), and we cannot rule out that VOs cryoinjuries are inherited by blastomeres after oocyte cleavage. Investigating features of embryos derived from either fresh or cryopreserved oocytes might help us to understand why embryo development progression is impaired in the latter, therefore it could suggest how to improve cat oocytes cryotolerance and pave the way to a more effective embryo production from VOs.

## Conclusions

Vitrification of immature cat oocytes induced an increase in DNA fragmentation and caspase activity, which could be reverted with the addition of the inhibitor Z-VAD-FMK during vitrification-warming and the following day of culture. The *in vitro* maturation potential and the fertilization rates of VOs treated with Z-VAD-FMK resulted similar to those of fresh oocytes, yet the inhibitor was not determinant for embryo development. The identification of specific alterations or activation of molecular pathways induced by vitrification in the intracellular environment of immature oocytes might suggest other strategies to inhibit cell death, enhance cat VOs cryotolerance, and improve their developmental abilities.

## Data Availability Statement

The raw data supporting the conclusions of this article will be made available by the authors, without undue reservation.

## Ethics Statement

Ethical review and approval was not required for the animal study because it used domestic cat gonads collected as byproducts from routine surgeries.

## Author Contributions

MC and JZ: conceptualization, methodology, and data curation. MC and SJ: investigation. MC: formal analysis and writing original draft. JZ: supervision. KJ and GL: conceptualization, resources, and funding acquisition. GL: project administration. All authors contributed to manuscript revision, read, and approved the submitted version.

## Conflict of Interest

The authors declare that the research was conducted in the absence of any commercial or financial relationships that could be construed as a potential conflict of interest.
